# A cluster of co-occurring cases of leptospirosis and brucellosis in a pastoral community of dairy farmers, India, 2023–2024: an epidemiological investigation

**DOI:** 10.5365/wpsar.2026.17.2.1327

**Published:** 2026-06-01

**Authors:** Farida Khatoon, Srividya K Vedachalam, Sushma Choudhary, Subodh Kumar Joshi, Pankaj Kumar Jain, Yogesh Bahurupi, Fareeduzzafar,, Meenu Singh, Pankaj Kumar Singh

**Affiliations:** a Epidemiology Division, National Centre for Disease Control, New Delhi, India.; b South Asia Field Epidemiology and Technology Network, New Delhi, India.; c Integrated Disease Surveillance Programme, Haridwar, Uttarakhand, India.; d All India Institute of Medical Sciences, Rishikesh, Uttarakhand, India.

## Abstract

**Objective:**

A cluster of leptospirosis and brucellosis cases in a semi-nomadic, pastoral community was reported in October 2023 from Village A, Uttarakhand State, India. This cluster was investigated to describe the epidemiology and potential exposures.

**Methods:**

Suspected cases were residents with sudden-onset fever or myalgia occurring from 1 August 2023 to 7 January 2024; confirmed cases were suspected cases with laboratory confirmation. Suspected cases were identified through a house-to-house case search and were interviewed regarding sociodemographic variables, symptoms and exposures. Those who consented were tested. Data were cleaned and analysed in Microsoft Excel.

**Results:**

Fifty suspected cases were identified among a total of 300 residents across 85 households. Among these, 27 agreed to be tested, and 25 were confirmed (13 with leptospirosis, 7 with brucellosis and 5 with both leptospirosis and brucellosis). The cases began in September 2023 following flooding and continued until January 2024, with no deaths. The median age (range) of cases was 30 (17–82) years for leptospirosis, 39 (25–56) years for brucellosis and 39 (22–50) years for coinfection. Altogether, females accounted for 92% (12/13) of leptospirosis cases, 57% (4/7) of brucellosis cases and 80% (4/5) of coinfections. Symptoms included fever, myalgia and arthralgia. All cases reported contact with floodwater and animal abortus.

**Discussion:**

This was a laboratory-confirmed co-occurring cluster of leptospirosis and brucellosis, including five cases of coinfection, following flooding, predominantly affecting female Van Gujjar dairy farmers. Education was provided to the community about the hygienic disposal of animal waste.

Leptospirosis, a bacterial zoonosis caused by *Leptospira* spp., has an estimated global burden of 2.9 million disability-adjusted life-years (DALYs) annually; South-East Asia has one of the highest burdens, with 137 DALYs per 100 000 population. (*1*) Brucellosis, caused by *Brucella* spp., another bacterial zoonosis, has an estimated annual incidence of 2 million cases, with three fourths of the cases in Asia. (*2*) Both diseases present with non-specific symptoms, such as fever, myalgia, malaise and headache, complicating diagnosis and treatment. (*3*, *4*) Pastoral communities are at risk for both these zoonoses because of their close contact with livestock. (*5*, *6*) However, few instances of simultaneous circulation of these pathogens in livestock and of human coinfection with both diseases have been reported, and none have been reported from India. (*7*, *8*)

The Van Gujjars are a forest-dwelling pastoral tribal community inhabiting the northern Indian Himalayan state of Uttarakhand. They follow a semi-nomadic lifestyle and migrate to the alpine regions of the western Himalayas along with their livestock during warmer months. (*9*, *10*)

In October 2023, a One Health camp (OHC) was piloted by the Department of Health in collaboration with the Department of Animal Husbandry in Uttarakhand. The OHC was conducted with the objective of screening high-risk populations for two zoonotic diseases: leptospirosis and brucellosis. The OHC screened the population of Village A, Bahadarabad Block, Haridwar District, Uttarakhand, in October 2023 and identified cases of leptospirosis and brucellosis within the village community. A follow-up investigation was carried out by the State Health Department in December 2023 to determine whether the cluster of cases identified during the OHC constituted an outbreak in Village A, to describe the epidemiology and potential exposures among cases, and to initiate prevention and control measures.

## Methods

A line list of leptospirosis cases was extracted from the Integrated Disease Surveillance Programme (IDSP) unit in Uttarakhand for 2020–2022 to determine the monthly outbreak threshold (calculated as the mean number of cases reported in 1 month from 2020 to 2022 + 2 standard deviations from the mean) and compared with the number of cases reported in 2023 to determine whether the reported cluster met the threshold for confirmation of an outbreak. Brucellosis is not a notifiable disease in the IDSP, so no historical data could be obtained for comparison with the number of reported cases in 2023.

Selected cases from the line list were interviewed using a validated semi-structured questionnaire (**Supplementary Material**). Based on the interviews and the IDSP case definitions of leptospirosis and brucellosis, definitions of suspected and confirmed cases were formulated for investigating this cluster, and additional cases were identified and interviewed.



### Case definitions

The definition of a suspected case of leptospirosis or brucellosis was the sudden onset of fever OR myalgia occurring from 1 August 2023 to 7 January 2024 in Village A. We used a broad, combined definition for suspected cases for both diseases because the initial patients in this community presented with these broad and non-specific symptoms. To capture all suspected cases, case-finding in the community started from 1 August 2023 (8 weeks, or two incubation periods, before the date of symptom onset in the index case, that is, 1 October 2023). In addition, to ensure that the investigation then went on to capture confirmed cases, we had a definition of a confirmed case that was specific for each disease.

The definition of a confirmed case of leptospirosis was a suspected case who tested positive by enzyme-linked immunosorbent assay (ELISA) for immunoglobulin M (IgM) for leptospirosis. The definition of a confirmed case of brucellosis was a suspected case who tested positive by ELISA for IgM for brucellosis. The definition of a confirmed case with coinfection with both leptospirosis and brucellosis was a suspected case who tested positive by ELISA for IgM for both leptospirosis and brucellosis.

### Case-finding strategy

The investigation used both active and passive case-finding strategies. For active case-finding, an investigation team comprising an Epidemic Intelligence Service officer from the National Centre for Disease Control, New Delhi, the Haridwar district surveillance officer, the medical officer in charge of the primary health centre and front-line health-care workers conducted house-to-house case-finding in Village A from 15 December 2023 to 7 January 2024, based on the case definitions formulated. Identified cases were interviewed to collect information about their sociodemographic details, clinical symptoms, health-care-seeking behaviour and exposure to floodwaters, cattle, animal abortus and raw animal products.

For passive case-finding, the investigation reviewed the records of the subcentre located in Village A to check for fever cases reported from 1 August 2023 to 7 January 2024.

### Laboratory investigation

Serum samples from suspected cases who consented to testing were sent to the district public health laboratory for ELISA analysis.

### Interviews with key stakeholders and field observation

Key stakeholders in the district human health sector, including front-line health-care workers and physicians at government health-care facilities, were interviewed to assess awareness of leptospirosis and brucellosis. During house-to-house case-finding, investigators observed each house’s surroundings, general sanitation and drinking-water sources.

### Data analysis

**Fig. 1:**
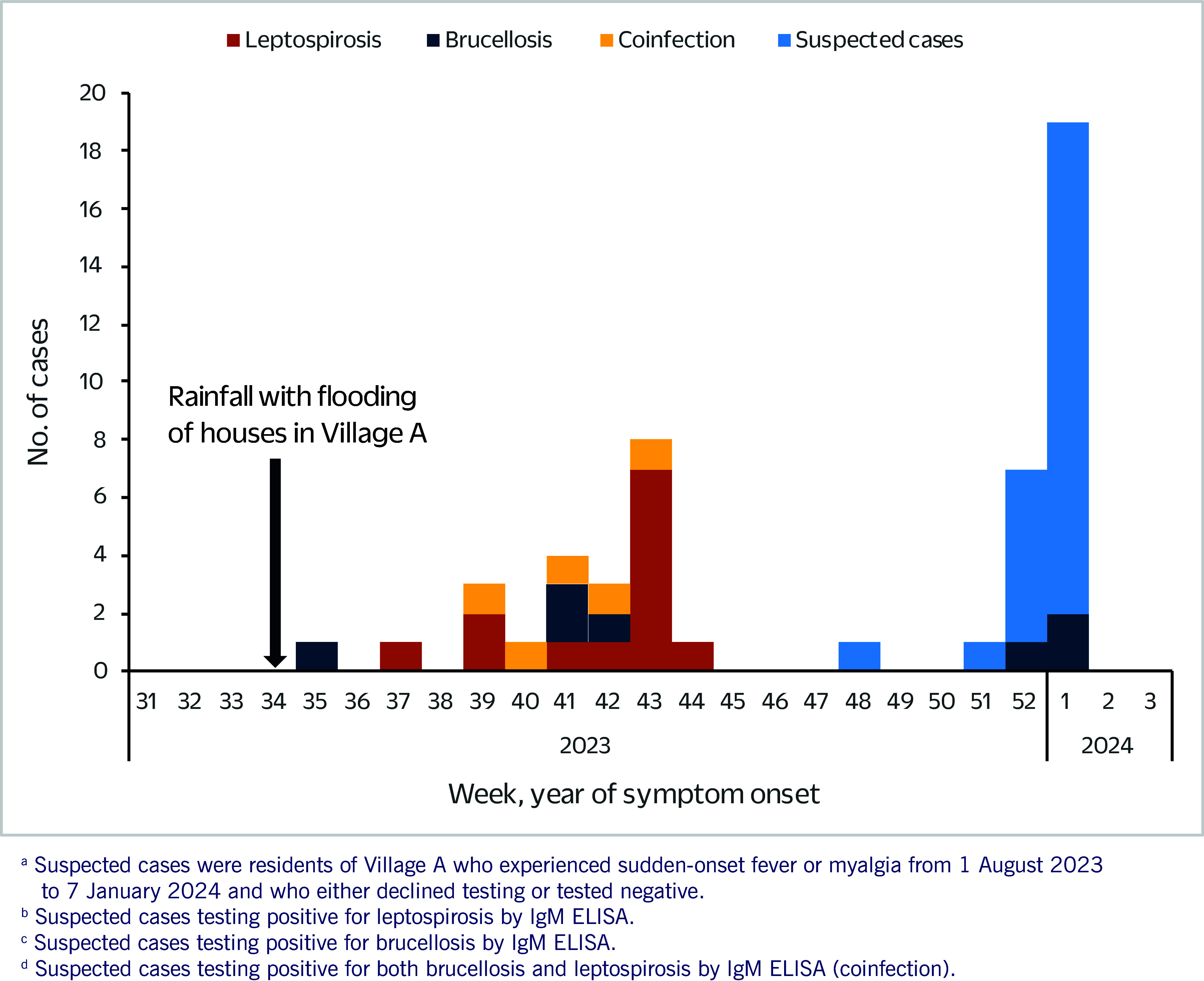
Epidemic curve of suspected^a^ (*N* = 25) and confirmed cases (*N* = 25) of leptospirosis,^b^ brucellosis^c^ and coinfection, ^d^ by week of symptom onset, Village A, Haridwar, Uttarakhand, India, 2023-2024

Continuous data on age were analysed to determine the median and range, and categorical data were expressed as frequencies and proportions using Microsoft Excel 2021. The distribution of cases by week of symptom onset was plotted on an epidemic curve (**Fig. 1**).

[**insert** Figure 1]

## Results

A total of 18 leptospirosis cases including five coinfected cases were identified, exceeding the annual number reported in each of the previous 5 years in Haridwar district (2018–2021, *n* = 0; 2022, *n* = 3). However, access to testing for leptospirosis at state and district levels was limited until 2021 (*n* = 35 tests), increasing in 2022 (*n* = 121) and 2023 (*n* = 632), with test positivity remaining the same (2%; data not shown). Brucellosis is not a notifiable disease in the IDSP, and this was the first OHC in the district; hence, no historical data for brucellosis could be obtained for comparison with the number of reported cases in 2023.

### Descriptive epidemiology

Village A had a population of 300 residents across 85 households. Active case-finding conducted among all residents identified 50 suspected cases, 27 of whom consented to testing for leptospirosis and brucellosis by IgM ELISA; the rest did not consent to testing. Among the 27 cases tested, 25 tested positive (confirmed cases): 13 for leptospirosis, 7 for brucellosis and 5 for both. The attack rate for leptospirosis including coinfections was 6% (18/300) and for brucellosis including coinfections was 4% (12/300). There were no deaths.

Among the 13 confirmed cases with only leptospirosis, the median age was 30 (range: 17–82) years, and 92% (12/13) were female. The most common symptoms were fever (92%, 12/13), myalgia (92%, 12/13) and arthralgia (85%, 11/13).

Among the seven confirmed cases with only brucellosis, the median age was 39 (range: 25–56) years, and 57% (4/7) were female. The most common symptoms were fever (86%, 6/7), myalgia (57%, 4/7) and arthralgia (43%, 3/7).

Among the five confirmed cases coinfected with both leptospirosis and brucellosis, the median age was 39 (range: 22–50) years, and 80% (4/5) were female. The most common symptoms were fever (100%, 5/5), myalgia (80%, 4/5) and arthralgia (80%, 4/5).

Among the 50 suspected cases, 25 were laboratory-confirmed, and the remaining 25 were suspected cases (as they either were not tested or tested negative). Among these remaining 25 suspected cases, the median age was 36 (range: 17–70) years, and 80% (20/25) were female. The most common symptoms were fever (76%, 19/25), myalgia (60%, 15/25) and arthralgia (48%, 12/25).

All cases (both suspected and confirmed) occurred among the Van Gujjars, who have a semi-nomadic lifestyle and engage in cattle rearing, dairying and crop farming as their primary occupation. All reported contact with floodwater, wastewater contaminated with animal urine, and close contact with cattle and rodents in the preceding month, and routinely handling animal abortus or their fluids with bare hands. All cases reported that they first contacted a private physician when seeking health care during their illness (**Table 1**). The cases started in the first week of September 2023 and continued until the first week of January 2024 (**Fig. 1**).

**Table 1:** Sociodemographic characteristics, symptoms, exposures and laboratory results of suspected and confirmed cases of leptospirosis, brucellosis and coinfection, Village A, Haridwar, Uttarakhand, India, 2023–2024

Characteristics	Suspected^a^ (*n* = 25)	Confirmed^b^ (*n* = 25)		
		Leptospirosis (*n* = 13)	Brucellosis (*n* = 7)	Coinfection (*n* = 5)
**Median (range) age, years**	**36 (17–70)**	**30 (17–82)**	**39 (25–56)**	**39 (22–50)**
**Sex**				
**Female**	**20 (80)**	**12 (92)**	**4 (57)**	**4 (80)**
**Occupation**				
**Cattle rearing and dairying**	**25 (100)**	**13 (100)**	**7 (100)**	**5 (100)**
**Farming**	**25 (100)**	**13 (100)**	**7 (100)**	**5 (100)**
**Type of dwelling**				
**Semi-permanent (mud-plastered walls with straw roof)**	**25 (100)**	**13 (100)**	**7 (100)**	**5 (100)**
**Cattle shed**				
**Attached to house**	**25 (100)**	**13 (100)**	**7 (100)**	**5 (100)**
**Educational status**				
**No formal education**	**7 (28)**	**6 (46)**	**3 (43)**	**2 (40)**
**Some formal education**	**18 (72)**	**7 (54)**	**4 (57)**	**3 (60)**
**First contact with a health facility seeking care after illness**				
**Private physician**	**25 (100)**	**13 (100)**	**7 (100)**	**5 (100)**
**Symptoms**				
**Fever**	**19 (76)**	**12 (92)**	**6 (86)**	**5 (100)**
**Myalgia**	**15 (60)**	**12 (92)**	**4 (57)**	**4 (80)**
**Arthralgia**	**12 (48)**	**11 (85)**	**3 (43)**	**4 (80)**
**Headache**	**0 (0)**	**9 (69)**	**1 (14)**	**4 (80)**
**Fatigue**	**0 (0)**	**8 (62)**	**3 (43)**	**2 (40)**
**Skin rash**	**0 (0)**	**0 (0)**	**1 (14)**	**0 (0)**
**Chills**	**0 (0)**	**0 (0)**	**5 (71)**	**0 (0)**
**Exposure factors (during month before symptom onset)**				
**Contact with floodwater**	**25 (100)**	**13 (100)**	**7 (100)**	**5 (100)**
**Contact with wastewater contaminated with animal urine**	**25 (100)**	**13 (100)**	**7 (100)**	**5 (100)**
**Close contact with cattle**	**25 (100)**	**13 (100)**	**7 (100)**	**5 (100)**
**Rodent sighting within the house**	**25 (100)**	**13 (100)**	**7 (100)**	**5 (100)**
**History of bathing in a common waterbody**	**0 (0)**	**0 (0)**	**0 (0)**	**0 (0)**
**Consumption of raw (unboiled) milk**	**0 (0)**	**0 (0)**	**0 (0)**	**0 (0)**
**Consumption of curd/butter made with raw (unboiled) milk**	**0 (0)**	**0 (0)**	**0 (0)**	**0 (0)**
**Consumption of raw, uncooked meat**	**0 (0)**	**0 (0)**	**0 (0)**	**0 (0)**
**Handling animal abortus or their fluids with bare hands**	**25 (100)**	**13 (100)**	**7 (100)**	**5 (100)**
**Positive laboratory results**				
**Leptospirosis**	**–**	**13 (100)**	**0 (0)**	**5 (100)**
**Brucellosis**	**–**	**0 (0)**	**7 (100)**	**5 (100)**

Values are *n*(%) unless otherwise indicated.

^a^ Suspected cases were residents of Village A who experienced sudden-onset fever or myalgia from 1 August 2023 to 7 January 2024 and who either declined testing or tested negative.

^b^ Confirmed cases were suspected cases who tested positive by IgM ELISA.

### Interview with key stakeholders

Six stakeholders were interviewed: one medical officer, two laboratory technicians, two village-level front-line health-care workers and one community leader. Front-line health-care workers had limited awareness of leptospirosis and brucellosis, and of their transmission from animals to humans. The physician was aware of the diseases but did not suspect they were present in the village.

### Field observations

Houses were made of mud-plastered brick walls and thatched roofs, with cattle sheds attached to them. All observed households had rodents. General sanitation of the area was poor, with accumulated wastewater around the houses seen contaminated with cow dung, and animal urine seen mixed with the accumulated wastewater. There was no municipal piped water supply, and the only source of drinking-water was a hand pump surrounded by wastewater. The community leader reported females being primarily engaged in rearing animals at home, while males travelled to the market to sell animal products. He also reported that residents initially sought health care from private physicians due to their accessibility and operational days (all days of the week). The community leader reported a history of pluvial flooding from September to October 2023, which was confirmed by news reports from the time.

## Discussion

A laboratory-confirmed mixed cluster of leptospirosis and brucellosis was identified, with simultaneous transmission of both, including a few cases of coinfection, in the Van Gujjar community residing in Village A of Bahadarabad Block, Haridwar District, Uttarakhand. The onset of cases followed flooding in the area. Fever, myalgia and arthralgia were the main symptoms. Most cases were female dairy farmers, mostly young and middle-aged, who presented with symptoms consistent with leptospirosis and brucellosis. (*3*, *4*) As adequate testing for leptospirosis only started in 2022, the investigation did not have enough evidence to determine whether this cluster of leptospirosis cases in 2023 was an outbreak or an increase in cases due to enhanced testing efforts.

Across the world, rural pastoral communities, similar to those in our investigation, are reported to be at a higher risk for bacterial zoonoses due to their close contact with animals, inadequate sanitation, poor access to health care or poor diagnostic capacity in these remote areas. (*11*-*13*) This risk is further complicated by the lack of awareness of these diseases among local health-care workers, resulting in missed or delayed diagnosis, which we documented in this investigation. The symptoms seen in these cases are consistent with those generally reported in brucellosis and milder cases of leptospirosis. However, the sex predilection generally reported in leptospirosis (80% males) is reversed in this investigation, with most cases occurring in females. (*1*, *14*) This might be due to higher occupational exposure of females compared to males in this community. Simultaneous transmission of both diseases in the community indicates a need to screen multiple zoonotic pathogens in at-risk communities.

The limitations of this investigation included the increase in testing during the preceding year, which hindered our understanding of whether this cluster was a true increase in cases. Additionally, in the absence of confirmatory testing for all suspected cases, patients with other febrile illnesses could potentially have been misclassified as cases of leptospirosis or brucellosis, leading to an overestimation of suspected cases.

### Public health action and recommendations

Following this investigation, the residents of Village A were informed by the investigation team of the need to drain animal urine away from common sources of accumulated water, improve general sanitation and initiate rodent control measures. Confirmed cases were referred for treatment at the nearest health facility. The investigation team also requested the waterworks department to provide a source of piped drinking-water to Village A. Health-care workers in the area were briefed on the symptoms of these diseases and on the need to test suspected cases.

The investigation team recommended that the state health department alert health-care workers to suspect leptospirosis in patients presenting with fever following rainfall and brucellosis among pastoralists.
