# Supplementary material for: A cluster of co-occurring cases of leptospirosis and brucellosis in a pastoral community of dairy farmers, India, 2023–2024: an epidemiological investigation
Source: Western Pac Surveill Response J. 2026 Jun 1;17(2):1–7. doi: 10.5365/wpsar.2026.17.2.1327 (PMC13382159; doi:10.5365/wpsar.2026.17.2.1327)
Supplement: Supplementary file 1 [file wpsar-17-1327-s001.pdf]

Supplementary Fig. 1. **Case Information Sheet**

Individual Code

Name: Age/Gender:

Religion: Caste:

Address: Phone number:

Area: Urban/Rural

1. Socialdemographic details

Occupation (in case of minor, occupation of head of family)	
Education status	
Total family members	
Total family monthly income	
Type of house	Kaccha (mud house)<br>Pucca (cemented house)<br>Mixed (part mud, part cemented)<br>Other: ________________
Number of rooms	
Is there a cattle shed in the house?	Yes<br>No
If yes, type of cattle shed	Kaccha (mud house)<br>Pucca (cemented house)<br>Mixed (part mud, part cemented)<br>Other
Is the cattle shed within the housing premises?	Yes<br>No
If no, distance of cattle shed from the premises (approx. in metres)	

2. Clinical details

Deceased	Yes/No
If yes: Details of interviewee	
Date of admission (triangulate from hospital records)	dd/mm/yyyy
Date of discharge	dd/mm/yyyy
Onset of symptoms (approx.)	Discharged/Left against advice/Death
Outcome	
Type of sample collected	
Date of sample collection	
Date of result	
Type of test done	
Test result	Positive<br>Negative<br>Other, please specify: ________________

Whom did you first consult?	Private physician<br>Government primary health centre<br>District hospital<br>Auxiliary nurse midwife<br>Accredited social health activist<br>Traditional healer<br>Other: ________________
Initial symptoms	Fever<br>Chills<br>Redness of eyes<br>Nausea<br>Vomiting<br>Abdominal pain<br>Headache<br>Body ache or generalized muscle pain<br>Pain and tenderness in the calf muscles Yellowish discoloration of eyes<br>Reduced urine output<br>Shortness of breath<br>Swelling of feet<br>Generalised oedema<br>No symptoms
Other symptoms	Nausea<br>Vomiting<br>Diarrhoea<br>Cough<br>Rash<br>Abdominal pain<br>Photosensitivity<br>Other: ________________
Complications, if any	Renal failure<br>Jaundice<br>Bleeding<br>Hepatic failure<br>Encephalopathy<br>Cardiac failure<br>Respiratory distress<br>Hypotension<br>Any other, please specify: ________________
Did you require ICU admission?	Yes<br>No
Comorbidities, if any	Hypertension<br>Diabetes<br>Tuberculosis<br>Thyroid disorder<br>Stroke<br>Chronic renal disorder<br>Chronic liver disease<br>Obesity<br>Cancer<br>Pregnancy<br>Other: ________________
Previous history of leptospirosis	Yes<br>No<br>Don't know
If yes, specify	

3. Exposure details (30 days before symptoms onset)

Date of symptom onset	dd/mm/yyyy
History of direct or indirect exposure with animals<br>(Explain indirect contact to the interviewee)	Yes<br>No<br>Don't know
If yes, type of animal	Cows<br>Buffalo<br>Goats<br>Sheep<br>Horses<br>Pigs<br>Dogs<br>Cats<br>Rodents<br>Others
Any specific animal contact	Detail of the event with the type of animal
Did you have any open wound/abrasions/cut during the exposure (based on recall)?	Yes<br>No<br>Don't know<br><br>If yes, details: ____________
Do you belong to any occupation with frequent animal contact?	Farmer<br>Veterinarian<br>Abattoir worker<br>Poultry farm<br>Others, please specify
Other modes of animal exposure, if any	Pet owner<br>Gardening<br>Animal rescuer<br>Animal researcher<br>Zoo<br>House rodents<br>Others, please specify: __________
Do you remember any contact with water source before illness?	Yes<br>No<br>Don't know
Do you belong to any of these occupations where water exposure is present?	Farmer<br>Marine industry<br>Fisherman<br>Coast guard<br>Swimming pool
History of any recreational exposure	Water sports<br>Boating<br>Swimming (public/private pool)<br>Camping<br>Bushwalking<br>Fishing

4. Exposure to infected water (30 days before symptoms onset)

Was there heavy rainfall near place of residence/work?	Yes<br>No<br>Don't know
Were there floods in the area of residence/work?	Yes<br>No<br>Don't know
History of consumption of untreated water	Yes<br>No<br>Don't know
Any contact with floodwater runoff or sewage	Yes<br>No<br>Don't know
History of bathing in irrigation water canal	Yes<br>No<br>Don't know
Practice of open defecation	Yes<br>No<br>Don't know

5. Exposure to animal products (for brucellosis)

Do you routinely consume milk?	Yes<br>No<br>Don't know
If yes, which animal's milk do you consume?	Cattle<br>Goat
Do you routinely boil milk before consuming?	Yes<br>No<br>Don't know
Do you routinely consume curd?	Yes<br>No<br>Don't know
Do you routinely boil milk before making curd?	Yes<br>No<br>Don't know
Do you routinely consume butter?	Yes<br>No<br>Don't know
Do you routinely boil milk before making butter?	Yes<br>No<br>Don't know
Do you consume raw meat?	Yes<br>No<br>Don't know
Do you handle animal abortus, if required?	Yes<br>No<br>Don't know
How do you handle animal abortus?	Bare hands<br>Gloved hands<br>Don't remember
In the last month, did you handle any animal abortus?<br>If yes, how?	

6. Other relevant history

History of travel	Yes<br>No<br>Don't remember<br>If yes, details:<br>Date of journey:<br>Place:<br>Duration of stay:<br>Any of the above exposure there, if yes, details: ______________
Any other case of similar illness in the family	

7. **Environmental survey:** Interviewer should conduct a thorough environmental and household survey for the presence of rodents/sanitation/standing water/flooding/cow dung storage in the house and mention the details here

8. **Notes**
